# Effectiveness of thiazide and thiazide-like diuretics in advanced chronic kidney disease: a systematic review and meta-analysis

**DOI:** 10.1080/0886022X.2022.2163903

**Published:** 2023-01-13

**Authors:** Flávio Teles, Jorge Artur Peçanha de Miranda Coelho, Rosivânia Maria Albino, Fernanda Cristina Verçosa Pacheco, Evilly Rodrigues de Oliveira, Marcelo Augusto Duarte Silveira, Audes Diógenes M. Feitosa, Rodrigo Bezerra

**Affiliations:** a School of Medicine, Federal University of Alagoas (UFAL), Maceió, Brazil; b School of Medicine, State University of Health Sciences of Alagoas (UNCISAL), Maceió, Brazil; c Center for Higher Studies of Maceió (CESMAC), School of Medicine, Maceió, Brazil; d D'Or Institute for Research and Education (IDOR), Hospital São Rafael, Salvador, Bahia, Brazil; e Pernambuco Hypertension Service (SHIP), PROCAPE - University of Pernambuco (UPE), Recife, Brazil; f Keizo Asami Laboratory of Immunopathology, Federal University of Pernambuco, Recife, Brazil

**Keywords:** Chronic kidney disease, hypertension, diuretics, thiazides

## Abstract

**Background and objective:**

Thiazide diuretics are first-line drugs for the treatment of hypertension, but hypertension treatment guidelines have systematically discouraged their use in patients with advanced chronic kidney disease (CKD). For the first time, a systematic review and random-effects meta-analysis were performed to assess the effectiveness of thiazides and thiazide-like diuretics to treat hypertension in patients with stages 3b, 4, and 5 CKD.

**Design, setting, participants, & measurements:**

A systematic review and random-effects meta-analysis that included a literature search using the following databases were performed: MEDLINE through PubMed, Cochrane Database of Systematic Reviews (CDSR) and Cochrane Central Register of Controlled Trials (CENTRAL) through the Cochrane Library, Embase, and ISI – Web of Science (all databases). Prospective studies that evaluated the effectiveness of thiazide and thiazide-like diuretics in individuals with a GFR < 45 mL/min/1.73 m^2^ were included.

**Results:**

Five clinical trials, totaling 214 participants, were included, and the mean GFR ranged from 13.0 ± 5.9 mL/min/1.73 m^2^ to 26.8 ± 8.8 mL/min/1.73 m^2^. There was evidence of a reduction in mean blood pressure and in GFR, as well as in fractional sodium excretion and fractional chloride excretion.

**Conclusion:**

Thiazide and thiazide-like diuretics seem to maintain their effectiveness in lowering blood pressure in patients with advanced chronic kidney disease. These findings should spur new prospective randomized trials and spark discussions, particularly about upcoming hypertension guidelines.

## Introduction

Thiazide and thiazide-like diuretics are among the most commonly used medications in clinical practice. Their action in blocking sodium transport in the distal tubule induces a secondary increase in potassium excretion and calcium reabsorption. This effective change in the tubular transport of these three ions makes this class of diuretics an excellent alternative in the treatment of hypertension, edematous states, nephrolithiasis, osteoporosis due to hypercalciuria, and in some cases, hyperkalemia [1].

At the beginning of its clinical use, the concept emerged that the efficacy of this medication would be compromised in patients with a glomerular filtration rate (GFR) less than 30 mL/min/1.73 m^2^ [2]. Since then, this concept has been accepted as unquestionable evidence, and this threshold has been used for years by textbooks and guidelines to contraindicate the use of thiazides in patients with advanced chronic kidney disease (CKD) [3,4]. In this respect, the 2017 consensus of the American College of Cardiology/American Heart Association (ACC/AHA) for the treatment of arterial hypertension recommended that thiazides should be avoided in patients with GFR < 30 mL/min/1.73 m^2^ [3]. Similarly, the 2018 ESC/ESH Guidelines for the management of arterial hypertension stated that thiazides and thiazide-like agents are less effective antihypertensive agents in patients with an eGFR < 45 mL/min/1.73 m^2^ and become ineffective when the eGFR is < 30 mL/min/1.73 m^2^ [5]. However, especially from the 1980s on evidence against this concept has been published, and more recently, relevant results in the reduction of cardiovascular risk have renewed interest in this class of diuretics in patients with CKD [6–8]. Restricting the use of thiazides to individuals with a GFR greater than 45 mL/min/1.73 m^2^ means that a considerable portion of the hypertensive population does not receive this treatment, which is currently considered first-line due to the significant impact on cardiovascular mortality [9].

Thus, the aim of this study was to critically analyze studies with the main objective of assessing the effectiveness of thiazide and thiazide-like diuretics to treat hypertension in patients with advanced CKD.

## Methods

The study protocol was registered in the International Prospective Register of Systematic Reviews (PROSPERO) and is available under registration number CRD42021238110 (https://www.crd.york.ac.uk/prospero/display_record.php?ID=CRD42021238110). Possible revisions to the current topic were also researched in PROSPERO, avoiding redundant efforts. The Preferred Reporting Items for Systematic Reviews and Meta-Analysis Protocols (PRISMA-P) and methods set up by the Cochrane Collaboration were used [10]. When data was given in a way that didn’t work for a meta-analysis, the authors who made the data were contacted.

### Inclusion criteria

The inclusion criteria were guided by the following research question: ‘In individuals with advanced CKD, is there effectiveness in therapy with thiazide diuretics compared to no treatment?’ which was formulated using the PICO strategy. The acronym ‘P’ (population) corresponds to individuals with CKD and GFR < 45 mL/min/1.73 m^2^; ‘I’ (intervention), to the use of thiazide diuretics; ‘C’ (comparison) refers to nontreatment, other diuretics, or antihypertensives; and ‘O’ (outcome) refers to the improvement of clinical and laboratory parameters such as: (1) the primary outcome: lowering blood pressure (BP); (2) the secondary outcomes: increase in fractional sodium excretion (FeNa^+^), fractional chloride excretion (FeCl^−^) and changes in GFR. Urinary output, body weight (BW), serum potassium, sodium and uric acid levels were also described but not included in the meta-analysis. Studies that did not focus primarily on thiazide efficacy testing in patients with advanced CKD or that did not present clinical or laboratory markers of the diuretic effect were excluded.

### Search strategy and selection process

The search strategy was performed using the following databases: MEDLINE *via* PubMed, Cochrane Database of Systematic Reviews (CDSR) and Cochrane Central Register of Controlled Trials (CENTRAL) *via* the Cochrane Library, Embase, and ISI – Web of Science (all databases). Two different authors (J.A.P.M.C. and F.T.) used Rayyan (https://rayyan.ai) with the blind option turned on to evaluate and choose the studies. If there was a disagreement, a third author (R.B.), who was more experienced, was available to help [11]. The searches were performed to identify completed studies from 1994 to October 2022. After this first step, we added the chosen papers to Research Rabbit (https://researchrabbitapp.com) so that it could automatically find other studies and check all of their references (see figure of connections between articles in supplemental material) [12]. The authors read titles and abstracts to verify that the study met the eligibility criteria. In the selection process, the following were excluded: (1) studies using animal models, observational studies, and nonsystematic reviews; (2) studies that included patients with stage ≥ 3a CKD (≥ 45 mL/min/1.73 m^2^). This threshold was chosen because 30–40 mL/min/1.73 m^2^ has been used in textbooks for years and 45 mL/min/1.73 m^2^ for important guidelines (2018 ESC/ESH Guidelines for the Management of Arterial Hypertension) to contraindicate the use of thiazides in patients with CKD; (3) studies that used only a group treated with the association of thiazides with other diuretics versus other antihypertensive drugs; (4) studies that did not assess the primary and two or more secondary outcomes. Only clinical prospective studies remained for the final sample.

### Data extraction and quality assessment

The data extraction process was performed by two authors previously trained (F.T. and M. A. D. S.) and independently. Afterwards, the extracted data were checked, and there were no inconsistencies. All data relevant to the study were extracted using a standardized form. Data included the name of the first author, the year of publication, the type of study, participant selection technique, the intervention group, the outcomes of clinical variables, such as type and dose of diuretics, duration of treatment, and change in BP levels, eGFR or measured GFR, fractional sodium excretion (FeNa^+^) and fractional chloride excretion (FeCl^−^), laboratory variables, such as serum potassium and uric acid, and a report of major side effects (hyponatremia, acute kidney injury). Details regarding the extracted data are available in the supplemental material. The clinical and laboratory variables described here were chosen because they show some of the ways that thiazides work and were therefore thought to be useful for judging how well they work. Data extraction for various variables was performed at baseline (without thiazides) and at the end of treatment with thiazides in studies with long-term protocols. In one study [13] with a short-term design (single intravenous infusion), data extraction was performed at baseline and when the drug reached its maximum effect. In this review, we used the Cochrane Risk of Bias in Non-Randomized Studies of Interventions (ROBINS-I) assessment tool since most studies were not randomized [14]. Two review authors, F.T. and R.M.A., independently used ROBINS-I on each of the included studies. When they disagreed, they talked about it or asked a third review author, R.B., for advice. The complete risk of bias assessment is provided in the supplemental material.

### Outcomes and statistical analyses

We performed a random-effects meta-analysis. The Variables included in the meta-analysis were mean BP, GFR, FeNa^+^, and FeCl^−^. Data was collected at two distinct points in time (before and after intervention), and its variance for the different outcomes was measured. We calculated the differences using the mean, standard deviation, and sample size [15]. One study did not provide the standard deviation [16] and in this case, we obtained this data from the confidence interval. Flisser et al. [13], Dussol et al. [17] and Dussol et al. [6] presented data on blood pressure only as means. On the other hand, Agarwal et al. [18] and Agarwal et al. [16] provided data on systolic and diastolic blood pressure. For this reason, we have expressed arterial blood pressure as mean. A classical meta-analysis was performed in Review Manager (software version 5.4). We used the Random-Effect Model, Q-test (suggests heterogeneity between studies when the *p*-value is less than 0.15.), *I*^2^ statistic estimates (greater than 50% suggests substantial heterogeneity), and tau [2] (tau [2] > 0 suggests heterogeneity).

## Results

### Study quality assessment

**Figure 1.**
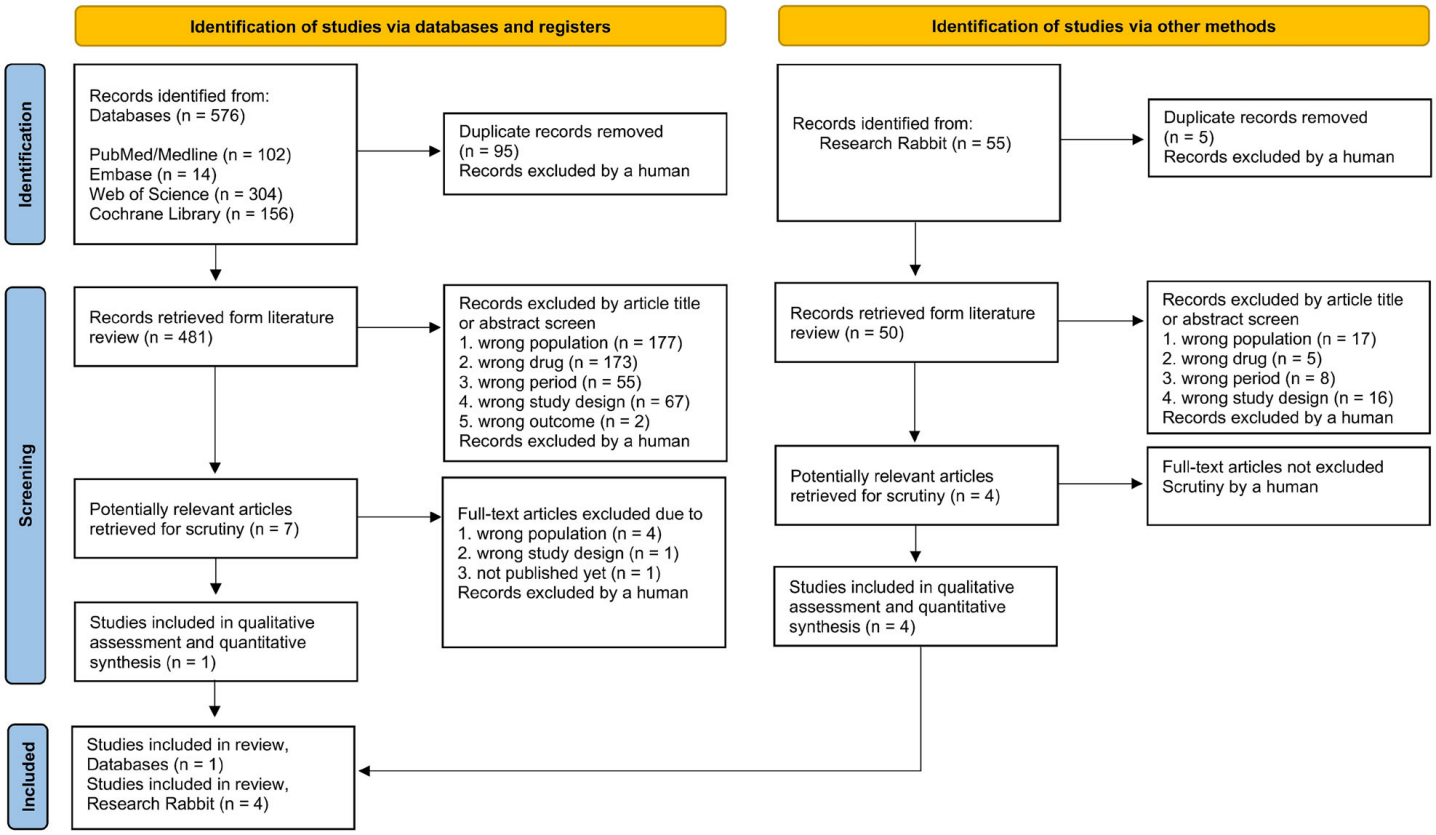
Flow diagram of the review process according to the Preferred Reporting Items for Systematic Reviews and Meta-analyses (PRISMA) statement.

There were 576 article abstracts found. The detailed flow diagram of the rationale for exclusion from the study is shown in Figure 1. After the exclusion process, a total of five articles remained in the final sample. These five studies analyzed a total of 214 patients and were published between 1994 and October 2022. The articles in the final sample were analyzed in detail. Flisser et al. [13], Dussol et al. [17], Dussol et al. [6], and Agarwal et al. [16] presented a low risk of bias. Agarwal et al. [18] presented a moderate risk of bias, due to a possible bias in measurement of outcomes and an open-label design.

### Studies characteristics and patient population

Table 1 summarizes the main characteristics of all eligible studies and their samples. Regarding study characteristics, hydrochlorothiazide was used in two studies, chlorthalidone in two studies, and butizide in one study. These studies either compared thiazides (and thiazide-like diuretics) to loop diuretics or compared monotherapies of thiazides and loop diuretics to the association between the two drugs. Flisser et al. [13] performed a randomized placebo-controlled trial that included ten patients with a mean GFR of 13.0 ± 5.9 mL/min/1.73 m^2^ and compared torasemide plus butizide versus torasemide plus placebo for 24 days [13]. Dussol et al. [17] performed a randomized double-blinded crossover trial that included seven patients with a mean GFR of 25.4 ± 10.0 mL/min/1.73 m^2^ and compared long-action furosemide versus hydrochlorothiazide and combined therapy for one month [17]. Dussol et al. [6] performed another randomized double-blinded crossover trial that included twenty-three patients with a mean GFR of 24.6 ± 13.0 mL/min/1.73 m^2^ and compared furosemide versus hydrochlorothiazide for three months [6]. Agarwal et al. [18] performed a single arm study without a control group that included fourteen patients with a mean GFR of 26.8 ± 8.8 mL/min/1.73m^2^ and compared a standard regimen of antihypertensives with the addition of chlorthalidone for 120 days [18]. Agarwal et al. [16] performed a randomized double-blinded placebo-controlled trial with 160 patients with a mean GFR of 23.2 ± 4.2 mL/min/1.73 m^2^ and used chlorthalidone by dose escalation every 4 weeks, up to 50 mg for 20 weeks [16]. Regarding the general patient population, all selected individuals were over eighteen years old. Patients had an average age ranging from 53.7 ± 7.8 to 67.5 ± 10.2 years and an average GFR ranging from 13.0 ± 5.9 mL/min/1.73 m^2^ to 26.8 ± 8.8 mL/min/1.73 m^2^. GFR was estimated (eGFR) in 2 studies and measured by inulin or DTPA in 3 others. Regarding the blood pressure measurement, Flisser et al. [13] performed five measures taken at a three-hour interval. Dussol et al. [17] and Dussol et al. [6] performed five measures taken at ten minutes interval. Agarwal et al. [18] and Agarwal et al. [16] performed 24-h home blood pressure monitoring. Regarding the etiology of CKD in the sample, diabetes was the most prevalent, and other causes included hypertensive nephrosclerosis, glomerulonephritis, polycystic kidney disease, obstructive uropathy, or an indeterminate etiology.

**Table 1.** General characteristics of the studies.

Primary author, year and country	Study design	Subjects and age	Inclusion criteria	GFR	Thiazide type and protocol	Main outcomes
Agarwal 2021, USA (CLICK trial)	Randomized, double-blinded, placebo-controlled trial	(*n =* 160) 66.2 ± 10.8	CKD stage 4 (eGFR < 30 and ≥ 15 mL/min) and hypertension (BP≥ 130/80 mmHg)	23.2 ± 4.2 ml/min/1.73 m^2^	Chlortalidone 12.5mg followed by dose escalation every 4 weeks, up to 50 mg. Total of 20 weeks. 60% also taking loop diuretic	Reduction of 11 mmHg in systolic blood pressure versus 0.5 mmHg in placebo group
Agarwal 2014, USA	Single arm without a control group	(*n =* 14) 67.5 ± 10.2	eGFR ≤ 45ml/min/ 1.73 m^2,^ and > 20 ml/min/1.73 m^2^ and hypertension (BP ≥ 135/85 mmHg)	26.8 ± 8.8 ml/min/1.73 m^2^	Chlortalidone 25 mg added to a standard regimen of other antihypertensives agents for 120 days	10.5 mmHg reduction in systolic BP likely evoked by lowering extracelular fluid volume (lowering of BNP and increase in renin and aldosterone concentrations)
Dussol 2012, France	Randomized, double-blinded crossover trial	(*n =* 23) 62 ± 13	CKD stage 4 or 5 (eGFR) and hypertension (BP > 130/80 mmHg)	24.6 ± 13 ml/min/1.73 m^2^	Furosemide 60mg versus hydroclorothiazide 25mg for three months	7% reduction of BP. Reduction on plasma potassium (0.5 mmol/L), increase in FeNa^+^ (14.7%) and FeCl^−^ (21%)
Dussol 2005, France	Randomized, double-blinded crossover trial	(*n =* 7) 54 ± 10	GFR< 40 ml/min and hypertension (mean arterial pressure 112 ± 11 mmHg)	25.4 ± 10 ml/min/1.73 m^2^	Long action furosemide 60 mg versus hydrochlorothiazide 25mg and combined therapy	11.6% reduction of BP. Increase in FeNa^+^ (32%) and FeCl^−^ (40%)
Fliser 1994, Germany	Randomized, single-blind, placebo-controlled crossover study	(*n =* 10) 53.7 ± 7.8	Inulin clearance lower than 30 ml/min and hypertension (mean arterial pressure 101 ± 12 mmHg)	13.0 ± 5.9 ml/min/1.73 m^2^	Torasemide 50 mg plus butizide 20 mg versus torasemide plus placebo for 24 days	Non-significant change in BP. Increase in FeNa^+^ (15.5%), FeCl^−^ (49%) and urinary output (9%)

### Random-effects meta-analysis

#### Mean blood pressure

**Figure 2.**
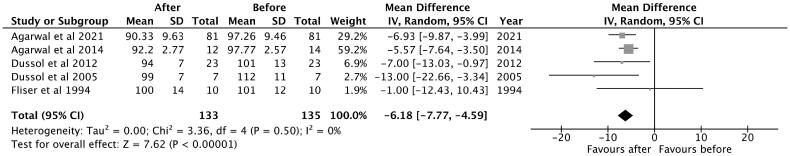
Mean difference of thiazide and thiazide-like diuretics in the mean blood pressure of patients with advanced CKD.

A total of five studies were included in the analysis. The observed mean difference was −6.18 (95% CI: −7.77 to −4.59) and the overall effect was *Z* = 7.62; *p* < 0.001. According to the Q-test, there was no significant amount of heterogeneity (Chi^2^ = 3.36, *p* = 0.50, tau [2] = 0.00, *I*^2^ = 0%) and no indication of outliers in the context of this model (Figure 2).

#### Glomerular filtration rate

**Figure 3.**
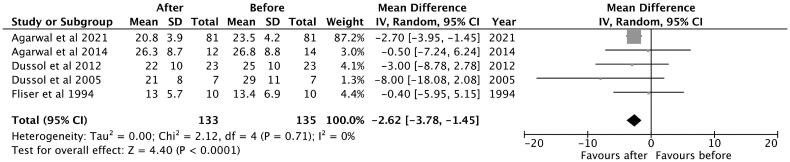
Mean difference of thiazide and thiazide-like diuretics in GFR of patients with advanced CKD.

A total of five studies were included in the analysis. The observed mean difference was −2.62 (95% CI: −3.78 to −1.45) and the overall effect was *Z* = 4.40; *p* < 0.001. According to the Q-test, there was no significant amount of heterogeneity (Chi^2^ = 2.12, *p* = 0.71, tau [2] = 0.00, *I*^2^ = 0%) and no indication of outliers in the context of this model (Figure 3).

#### Fractional sodium excretion

**Figure 4.**
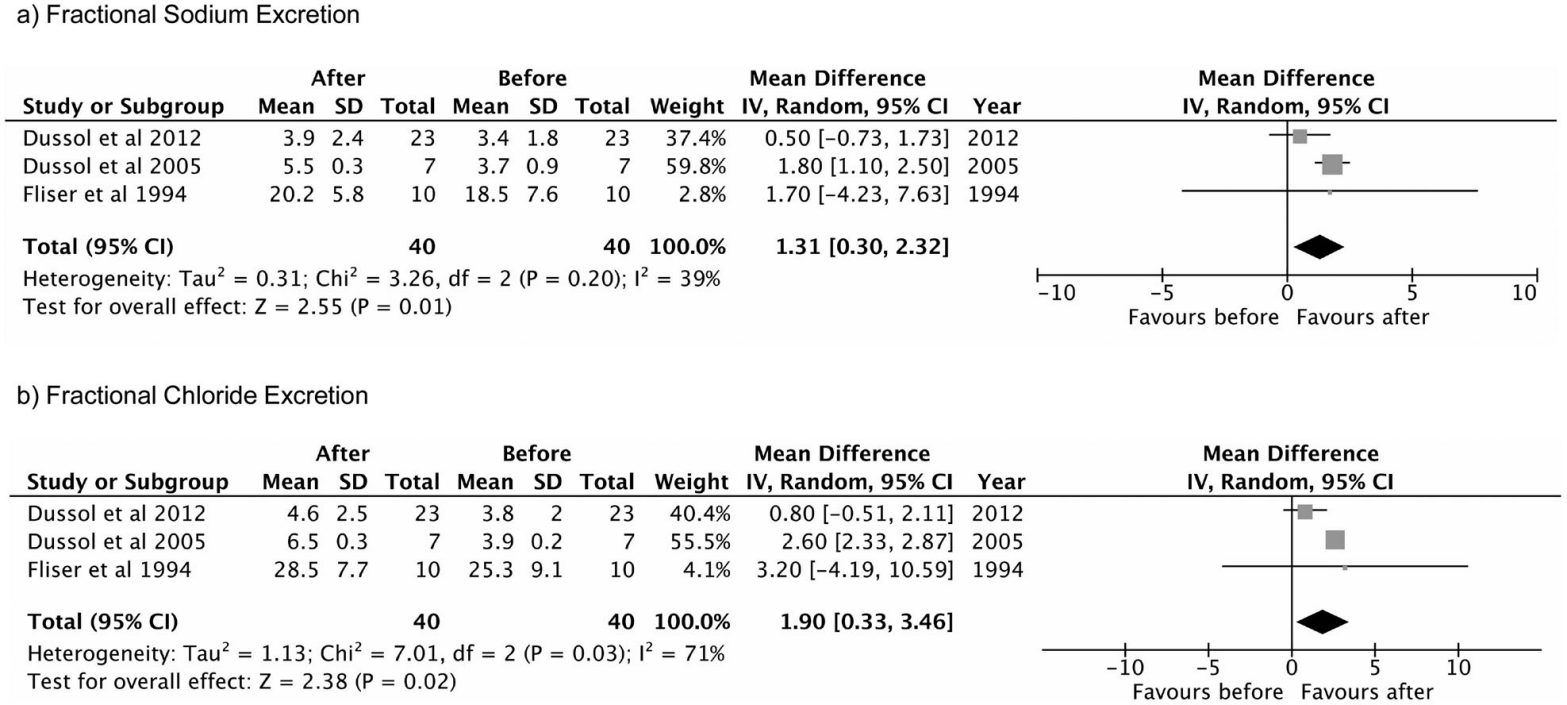
Mean difference of thiazide and thiazide-like diuretics in the (a) FeNa^+^ and (b) FeCl^−^ in patients with advanced CKD. (a) Fractional Sodium Excretion. (b) Fractional Chloride Excretion. As mean differences of (a) FeNa^+^ and (b) FeCl^−^ had positive values, ‘favours after’ was expressed in the right side.

Only three studies reported this variable and were included in the analysis. The observed mean difference was 1.31 (95% confidence interval: 0.30–2.32), and the overall effect was *Z* = 2.55, *p* = 0.01. According to the Q-test, there was no significant amount of heterogeneity (Chi^2^ = 3.26, *p* = 0.20, tau [2] = 0.31, *I*^2^ = 39%) and no indication of outliers in the context of this model (Figure 4(a)).

#### Fractional chloride excretion

Only three studies reported this variable and were included in the analysis. The observed mean difference was 1.90 (95% confidence interval: 0.33–3.46), and the overall effect was *Z* = 2.38, *p* = 0.02. According to the Q-test, the true outcomes appear to be heterogeneous (Chi^2^ = 7.01, *p* = 0.03, tau [2] = 1.13, *I*^2^ = 71%) (Figure 4(b)).

### Overall description of secondary outcomes

#### Urinary and serum potassium

Four of the five studies analyzed urinary potassium excretion, and all of them showed no significant change in this variable. Three studies assessed serum potassium levels. Dussol et al. [17] showed no significant reduction in serum potassium (basal state 4.7 ± 0.5 mmol/L versus 4.5 ± 0.7 mmol/L after hydrochlorothiazide). The same author in 2012 demonstrated a significant reduction in serum potassium after thiazide treatment (4.9 ± 0.7 mmol/L versus 4.4 ± 1.2 mmol/L after hydrochlorothiazide; *p* < 0.05), an effect like that observed with furosemide (basal 4.9 ± 0.7 mmol/L versus 4.5 ± 0.7 mmol/L after treatment; *p* < 0.05). Agarwal et al. [16, 18] didn’t find any big changes in potassium levels after treatment, only a few cases of mild hypokalemia.

#### Serum uric acid

Four studies evaluated variations in serum UA levels as an undesirable effect of thiazides. In all of the studies, an increase in UA was demonstrated, ranging from 1.0 to 2.2 mg/dL. Dussol et al. [17] demonstrated a nonsignificant increase (basal state 7.3 ± 2.2 mg/dL versus 9.5 ± 3.1 mg/dL after hydrochlorothiazide). In 2012, the same author found a significant increase in UA with hydrochlorothiazide (basal 7.1 ± 1.7 mg/dL versus 8.1 ± 2.1 mg/dL after treatment; *p* < 0.05). Agarwal et al. [18] demonstrated a significant increase in UA after 4 weeks of chlortalidone (mean increase of 1.64 mg/dL; *p* < 0.001). The same author observed hyperuricemia in 20% of patients treated with chlortalidone but only 2% with acute gout.

#### Serum sodium

All studies assessed serum sodium levels before and during the use of thiazides. Agarwal et al. [18] described mild hyponatremia in the first 8 weeks of treatment in a small portion of the sample (mean reduction of 2.0 mmol/L) and normalization at 12 weeks, with similar results in 2021. Dussol et al. [17] did not observe significant changes in serum sodium (140.0 ± 2.0 mmol/L versus 138.0 ± 3.0 mmol/L after hydrochlorothiazide). The same author found similar results in 2012 (141.0 ± 3.0 mmol/L versus 139.0 ± 4.0 mmol/L after hydrochlorothiazide).

#### Urinary output and body weight

Fliser et al. demonstrated a significant increase in urinary output with the use of a loop diuretic plus butizide versus a loop diuretic plus placebo (*p* < 0.05), but the other studies did not show a significant increase in this variable. Regarding BW, Agarwal et al. [18] showed a significant reduction in this parameter (1.7 kg) after 8 weeks of chlorthalidone (*p* = 0.05). Dussol et al. [17] observed a nonsignificant change in BW (85.0 ± 10.0 kg versus 84.0 ± 9.0 kg after hydrochlorothiazide). In 2012, the same author reported similar results. Agarwal et al. [16] observed a difference from baseline in BW in the chlorthalidone group versus the placebo group (–1.9 kg versus − 0.2 kg; CI −2.8 to −1.4).

## Discussion

In this random effects meta-analysis of clinical trials, we observed a difference in mean BP, suggesting that the antihypertensive action of thiazides is preserved, even in patients with advanced CKD. Regarding the risk of bias, it was generally low in most studies. Due to a an open-label design [18], had a moderate risk. In experimental models of CKD, a reduction in the reabsorption of NaCl and water by the proximal tubule has already been shown to cause a greater supply of these substances to the more distal segments of the nephron [19]. Blocking sodium transport with thiazides would explain the positive impact on the control of hypertension even in CKD. In the present study, we observed a difference in FeNa^+^ and FeCl^−^ that favored the treatment in the meta-analysis. However, these results must be interpreted with some caveats, as only three studies analyzed these variables, and only one [17], with a small number of participants, markedly favored the treatment, and hence influenced the final result. Nonetheless, some studies have demonstrated an effect of thiazides directly on the vessel wall, reducing peripheral vascular resistance, which could explain the maintenance of the antihypertensive effect at more critical levels of GFR [20].

Since the beginning of the use of thiazides, the concept has emerged that thiazides would be ineffective in patients with a GFR < 30 mL/min/1.73 m^2^. The origin of this concept was the study of Reubi and Cottier in 1961 [1]. In this study, 11 patients with variable stages of CKD received chlorothiazide. Of the eleven patients, six had a GFR greater than 50 mL/min/1.73 m^2^, and the others had a GFR less than 37 mL/min/1.73 m^2^. In the three patients with GFR values of 22, 32 and 37 mL/min/1.73 m^2^. FeNa^+^ increased approximately 2.5-fold after chlorothiazide. A similar effect was observed for the urinary excretion of potassium and chloride, as well as an important increase in urinary output. In the two patients with lower GFR (11 and 6 mL/min/1.73 m^2^), the effect of chlorothiazide on urinary FeNa^+^ was more modest, with an increase of approximately 1.5-fold. It is important to note that only three patients had a GFR less than 30 mL/min/1.73 m^2^ in this study, which makes the possibility of determining a threshold for ineffectiveness unlikely. Despite the fact that this study was performed before the era of the evidence-based medicine, its results have been cited in textbooks, for decades, stating that thiazides should not be used in patients with a GFR less than 30 mL/min/1.73 m^2^ [2]. But in the years since, evidence from observational studies and randomized trials has pointed to a different view [13,16,17].

Current methods of assessing kidney function have a ‘glomerulocentric’ view and exclusively take GFR into account. However, approximately 20% of the renal plasma flow is filtered, and the remaining 80% continues its way through the peritubular capillaries. At this point, solutes and drugs begin to interact with transporters present on the basolateral surface of the proximal tubule and initiate one of the most important processes of renal clearance: tubular secretion [21,22]. Thiazides have very limited glomerular filtration and reach their site of action through organic anion transporters (OAT1) present in the proximal tubule [23]. In people with advanced CKD, substances that have renal clearance by tubular secretion build up at a higher concentration than substances cleared by glomerular filtration [24]. Another important thing to note is that in some kidney diseases, the level of tubulointerstitial damage is not related to the level of glomerular damage, and as glomerular filtration goes down, tubular secretion goes up as an adaptive response [25,26]. Therefore, the exclusive use of the GFR calculation to assess the clearance of compounds with preferential elimination by tubular secretion can be quite imprecise. Chapron et al. recently showed that the estimated GFR and the effective renal elimination of several medications are very different, which supports this idea [27].

Thiazides are part of the therapeutic arsenal used to treat hypertension, standing out as one of the first-line choices. However, the main hypertension guidelines do not recommend thiazides in stages 4 and 5 CKD. For example, in the 2018 ESC/ESH Guidelines for the Management of Arterial Hypertension, it is recommended that thiazides be avoided in patients with a GFR < 45 mL/min/1.73 m^2^, giving preference to loop diuretics [5]. It is important to at least discuss the impact of changing this recommendations based on the following reasons. Loop diuretics have a shorter half-life (6 h) than thiazides, such as chlorthalidone. Given their longer half-life (12–24 h), thiazides ensure longer-lasting BP control with a lower risk of intravascular depletion compared with loop diuretics.

CKD is now considered a worldwide public health problem, and hypertension is its second most frequent cause. Thus, restricting the use of thiazides to individuals with a GFR greater than 40 mL/min/1.73 m^2^ means that a considerable portion of the hypertensive population does not receive this treatment. Therefore, based on the most recent evidence, guidelines, such as those proposed by the KDIGO Work Group, do not agree with the ACC/AHA and ESC/ESH recommendation to avoid thiazides in advanced CKD [28]. An additional finding in the present study was the significant mean difference in GFR with thiazides in this population of patients with advanced CKD, most of whom also used loop diuretics. Despite the reduction in GFR, there was no description of severe forms of acute kidney injury. The decrease in GFR could be caused by a drop in glomerular hydraulic pressure, which has already been shown in experiments with rats that had advanced CKD and were being treated with hydrochlorothiazide [29]. Furthermore, drugs with a well-established nephroprotective effect, such as angiotensin-converting enzyme inhibitors and angiotensin receptor blockers, and more recently SGLT2i can also reduce glomerular filtration in a transient way but with a long-term beneficial result. However, we can only speculate, and long-term studies will be needed to better clarify this issue.

In the present meta-analysis, no clinical trial showed an elevated frequency of serious side effects, most of them at standard doses. Agarwal et al. [18] demonstrated a higher frequency of nonserious events (hypokalemia, mild hyponatremia, hyperuricemia, small increases in creatinine, and orthostatic hypotension) with the use of higher doses of chlorthalidone (25–100 mg/day) [18]. Standard doses of thiazides are preferred in advanced CKD based on the following findings: (1) the excretion of thiazides is reduced in advanced CKD; 2) the use of higher doses, such as 50 mg per day of hydrochlorothiazide, can induce dehydration and hypokalemia, even in patients with CKD [30,31]. But side effects weren’t part of our main results, so we can’t say much about this topic in general.

## Limitations

This study has limitations, such as the use of different types of thiazides with distinct clinical protocols and levels of effectiveness, the small sample size of some studies, most of them not adequately powered, which might lead to lower odds ratio and tend to show more extreme treatment effects than larger ones. Furthermore, there was a lack of more robust data on side effects. However, for the first time, we used a random effects model to analyze thiazides’ effectiveness in advanced CKD.

## Conclusions

In this random effects meta-analysis, we demonstrate the existence of results that strongly raise the hypothesis that thiazide and thiazide-like diuretics would be effective in reducing blood pressure, even in patients with stage 4 and 5 CKD, and that the threshold of 30 or 40 mL/min/1.73 m^2^ for effectiveness is no longer justifiable. These findings should spur new randomized clinical trials and spark a broader debate about upcoming guidelines for the management of hypertension and CKD, so that the recommendations are based on more current and robust evidence and do not restrict the use of a drug with a relevant impact on such important outcomes.
